# Considering Histologic Remission in Ulcerative Colitis as a Long-Term Target

**DOI:** 10.3390/jcm13010289

**Published:** 2024-01-04

**Authors:** Christopher Pavel, Mircea Mihai Diculescu, Alex-Emilian Stepan, Gabriel Constantinescu, Vasile Sandru, Cristian George Ţieranu, Luiza Tomescu, Alexandru Constantinescu, Cristina Patoni, Oana-Mihaela Plotogea, Madalina Ilie

**Affiliations:** 1 Department of Gastroenterology, “Carol Davila” University of Medicine and Pharmacy, 050474 Bucharest, Romania; christopher.pavel@gmail.com (C.P.); mmdiculescu@yahoo.com (M.M.D.); gabrielconstantinescu63@gmail.com (G.C.); drsandruvasile@gmail.com (V.S.); cristian.tieranu@umfcd.ro (C.G.Ţ.); alexandruconstantinescu1991@gmail.com (A.C.); patonicristina@gmail.com (C.P.); drmadalina@gmail.com (M.I.); 2 Department of Gastroenterology, Clinical Emergency Hospital of Bucharest, 014461 Bucharest, Romania; 3 Department of Gastroenterology, Fundeni Clinical Institute, 022328 Bucharest, Romania; 4 Department of Pathology, University of Medicine and Pharmacy of Craiova, 200349 Craiova, Romania; astepan76@yahoo.com; 5 Department of Gastroenterology, “Elias” Emergency University Hospital, 011461 Bucharest, Romania; 6 Department of Pathology, “Carol Davila” University of Medicine and Pharmacy, 050474 Bucharest, Romania; luizaa.tomescu@yahoo.com; 7 Oncology Institute “Prof. Dr. Alexandru Trestioreanu”, 022328 Bucharest, Romania; 8 Department of Gastroenterology, Emergency University Hospital, 050098 Bucharest, Romania; 9 Department of Gastroenterology, Central Military Emergency Hospital “Dr. Carol Davila”, 010825 Bucharest, Romania

**Keywords:** ulcerative colitis, histologic scores, remission

## Abstract

Monitoring disease activity in inflammatory bowel disease (IBD) is challenging since clinical manifestations do not represent reliable surrogates for an accurate reflection of the inflammatory burden. Endoscopic remission had been the most significant endpoint target in the last years; nevertheless, a remarkable proportion of patients continue to relapse despite a normal-appearing mucosa, highlighting that endoscopy may underestimate the true extent of the disease. A subtle hint of the importance that histology plays in the long-term course of the disease has been endorsed by the STRIDE-II consensus, which recommends considering histologic healing for ulcerative colitis (UC), even though it is not stated to be a compulsory formal target. It is a continuum-changing paradigm, and it is almost a certainty that in the near future, histologic healing may become the new formal target for ulcerative colitis. It must be emphasized that there is great heterogeneity in defining histological remission, and the main criteria or cut-off values for inflammatory markers are still in an ill-defined area. The complexity of some histologic scores is a source of confusion among clinicians and pathologists, leading to low adherence in clinical practice when it comes to a homogenous histopathological report. Therefore, a standardized and more practical approach is urgently needed.

## 1. Introduction

Ulcerative colitis is a chronic inflammatory disease with a varying disease course, still bearing a high risk of surgery at 1, 5, or 10 years after diagnosis, despite a temporal decline in the need for surgery over the last six decades [1]. The main incriminated factor behind these relapses and the need for surgery is the persistent histological inflammation, which also translates into corticosteroid dependency, refractory patterns and a wide spectrum of complications leading up to the fulminant episodes. On the contrary, histologic remission in UC proved to determine an increased proportion of symptom-free patients [2], reduced the risk of relapse [3], hospitalizations [4] and colorectal cancer [5].

Since the 1980s, the main objective of clinical trials was the improvement of symptoms. In the following years, cumulative evidence led to the recommendation of some composite scores consisting of symptom resolution and mucosal healing as the primary endpoint. [6]. In UC, patients with a Mayo Endoscopic Score (MES) ≥ 1 have a threefold risk of clinical relapse compared with MES 0 [7]. However, mucosal healing does not always translate into histologic remission since an apparently normal mucosa may have persistent inflammatory activity. It was previously observed that up to 30% of patients with endoscopic remission do not achieve histologic remission [8]. Delving even deeper, previous data from our group identified mucosal gene transcripts associated with persistent histologic inflammation in UC patients who have achieved mucosal healing on endoscopy [9]. 

In 2007, D’Haens was one of the first authors to highlight histologic remission’s importance by stating that it should be strongly considered as a secondary endpoint in clinical trials [10]. There have been multiple attempts regarding the proper definition of histologic remission. According to the European Consensus on the histopathology of inflammatory bowel disease, remission is characterized by the resolution of the crypt architectural distortion and the inflammatory infiltrate, but some features of sustained damage may still be present, such as a decreased crypt density with branching and crypt shortening [11]. 

Since 2007, many studies have proved the added benefit of histologic remission in terms of lower relapse rate, cancer risk [11] and decreased hospitalization [12]. However, as clearly stated in the STRIDE-II consensus [13], histologic remission is challenging to obtain, and the required therapies necessary to achieve this objective might be a high burden from the perspective of adverse events and costs, with no similar benefit for every patient (i.e., younger vs. older age, pancolitis vs. proctitis, etc.). 

Furthermore, there is a lack of a standardized reporting method for histologic remission, which limits its clinical utility in daily practice. As demonstrated by Lemmens et al., there is a high degree of interobserver variability in histopathology reports, except for the extremes of the spectrum, i.e., inactive or severely active disease [14]. According to Calafat et al., defining histologic remission as the absence of intraepithelial neutrophils is appropriate and seems to be sufficient for prognostic purposes [15]. Another advantage of this approach is that the presence of neutrophils in the epithelium is the histological feature with the highest interobserver variability [16]. The importance of calprotectin serial measurements should not be forgotten as it was shown that elevated levels are correlated with persistent histologic activity, even in patients with macroscopic normal appearance (i.e., mucosal healing) [17]. Thus, in this particular scenario, we firmly believe the clinician must proceed with biopsies. 

With this consideration in mind, we should accept the real-world struggles of proper histopathology score knowledge and proper reporting. In a recent survey from Australia, only around 10% of pathologists reported the use of a systematic histologic scoring system, and more than 80% lacked awareness regarding the definition of remission according to different scoring systems [18]. 

The aim of this review is to scrutinize the available data regarding the most recent and validated histologic scores developed for ulcerative colitis and the individual subcomponents to determine the optimum standardized and practical approach. 

## 2. Parameters of Histologic Disease Activity

According to current guidelines [19], at least two biopsies from each of the five segments of the colon and from the terminal ileum should be taken at the index examination in every patient who has ulcerative colitis as a potential diagnosis. 

The normal cellularity of the intestine is not homogeneous in all segments (i.e., eosinophils are more prominent in the left colon), and it is important to send biopsies in different vials in order to properly assess the microscopic disease activity. UC can be characterized from a histologic point of view as chronic inactive, chronic active, or active (Table 1) [20]. 

The main microscopic features of IBD that should be highlighted in every histopathology report can be subdivided into features of activity and chronicity, respectively. Features of activity are represented by neutrophilic (or eosinophilic) cryptitis, crypt abscesses, necrosis, regenerative and degenerative epithelial changes, erosions, and ulcers [21]. Features of chronicity are represented by distorted crypt architecture (atrophy, foreshortening, irregular spacing, and/or size of crypts), basal plasmacytosis, basal lymphoid aggregates, lymphoplasmacytic infiltrate within the lamina propria or pyloric gland metaplasia [21]. 

### 2.1. Neutrophils

The landmark feature of active disease in UC patients is the presence of neutrophils within the lamina propria, surface epithelium, crypt epithelium, or in the lumens of crypts. As proven by Narula et al., the improvement of neutrophils in the epithelium was the only histologic parameter associated with increased odds of endoscopic and histologic remission at week 52 after induction therapy with Vedolizumab or Adalimumab [22]. Both neutrophils and epithelial damage should be used as a parameter since the sole presence of neutrophils might be induced by artifacts secondary to bowel preparation [23]. 

### 2.2. Eosinophils

The role of eosinophils is not so well established, but an abundant presence has been observed in ulcerative colitis, mostly in the initial phases of the disease and more frequently in children. There is also data that has linked persistent eosinophilic presence to an increased risk of relapse [24]. High eosinophilia is also associated with more severe colitis, a higher need for colectomy and a higher risk of primary sclerosing cholangitis [25]. On the contrary, the potential protective role of eosinophils was brought into discussion by a study that reported an even larger number of activated eosinophils during inactive UC [26]. Further research conducted by Isobe et al. revealed complex mechanisms that support the latter study, revealing that eosinophils can block PMN infiltration and modulate macrophage phenotype through cytokine mediators [27].

### 2.3. Basal Plasmacytosis

Basal plasmacytosis, defined as the infiltration of plasma cells extending into the lower one-third of the lamina propria, should be used both as a diagnostic feature and as a label of disease activity. It is linked to relapse of the disease in patients with complete mucosal healing, and the density correlates well with other histological features of disease activity [28].

### 2.4. Other Histologic Parameters

Other important active inflammatory parameters are the presence of mucosal ulcerations, erosions, mucin depletion and Paneth cell metaplasia. 

Mucin depletion, defined as a reduction in the number of goblet cells, is due to epithelial cell damage and can be recognized with good reproducibility. Severe-to-complete mucin depletion is a specific feature differentiating UC from Crohn’s disease. In a study that enrolled more than 190 UC patients, the presence of mucin depletion in patients with endoscopic remission (MES 0) was the only factor significantly and independently associated with the risk of relapse [29].

## 3. Histologic Scores in UC

Currently, there is no universally accepted method of grading histologic activity in biopsy specimens from patients with UC. The pioneer of histologic disease grading was Truelove, who described the mucosal alterations after hydrocortisone treatment in patients with ulcerative colitis in 1955 [30]. Thereafter, around thirty scoring systems were developed to assess histologic activity. Still, they are mainly used in research protocols, and their practical relevance in deciding how to manage patients’ therapy is limited [31]. Only a few underwent formal content validation and using non-validated scoring systems may have hindered the development of a systematic microscopic response to treatment in UC. 

Generally, histologic scores represent a combination of both acute and chronic changes focusing on inflammatory features and their effects on epithelial integrity. A validated and consistent scoring system is needed for international use, but currently, no such score exists. One major challenge is represented by the interobserver agreement, an issue that may limit the implementation of routine histologic remission as a treatment target in ulcerative colitis. One study found satisfactory inter-observer concordance for histological indexes. However, there was a poor correlation between primary histological assessment and reassessment of colonic biopsies by a second pathologist with expertise in IBD [32]. 

The three scoring methods proposed include the Geboes score and the Nancy and the Robarts histopathological indexes; the former is one of the most widely known and consecrated, and the latter two are the most validated. 

### 3.1. Geboes Score

The Geboes score (GS) was conceptualized more than 20 years ago as a histologic activity system that showed good reproducibility but modest agreement with the endoscopic grading system [33]. It has been used in numerous clinical trials, including new biologics molecules, even though it has never been validated. The Geboes score evaluates seven histological features of inflammatory bowel disease, but due to its complexity, it never adhered to daily clinical practice. The histologic features are represented by:

- Architectural changes (grade 0);
- Chronic inflammatory infiltrate (grade 1);
- Presence of eosinophils (grade 2A) or neutrophils (grade 2B);
- Neutrophils in epithelium (grade 3);
- Crypt destruction (grade 4);
- Erosions and ulcerations (grade 5).

Each grade is further divided into four or five subgrades that are evaluated by considering the most affected area of the biopsy. The GS can be used by assigning a score from 0 to 6 (the highest GS subscore seen in the biopsy) or in a continuous manner (scores from all GS subscores are summed and generate the continuous GS score) [34].

Histologic remission is defined as continuous GS ≤ 6 or GS ≤ 2 (absence of epithelial neutrophils) [34].

A simplified version, the Simplified Geboes Score, was proposed in 2016, and the assessments of histologic activity based on the two scores were comparable [35]. 

### 3.2. Robarts Histopathological Index

The Robarts Histopathological Index (RHI) was developed in 2017, and it is mainly derived from the GS. It includes four items that showed excellent inter-rater and intra-rater reliability, and each one receives a grade from 1 to 4:

- Lamina propria chronic inflammation;
- Lamina propria neutrophils;
- Epithelial neutrophils;
- Surface epithelial injury.

Histologic remission is defined as RHI < 3, while histological response is defined as RHI < 9. 

Even though RHI can be considered a simplified version of the GS, an analysis of three prospective cohorts showed that both scores are strongly correlated in their definitions of histological response, as 95% of patients classified as having histological response according to RHI also do so by the same criteria of the GS [36]. 

### 3.3. Nancy Index

The Nancy Index (NI) analyzes three characteristics of mucosal activity: acute, chronic inflammatory infiltrate and ulceration. Assessment of ulceration is made by the presence or absence of ulceration, in contrast to grading ulceration, which was used by GS [37]. With reference to NI, it was found that using only three grades of acute inflammatory cell infiltrate (absent, mild, or moderate to severe) was sufficient, while assessment of basal plasmacytosis as an index of chronic inflammatory infiltrate did not improve the index’s sensitivity, being therefore considered futile [37]. This stepwise evaluation based on the worst feature of each of the three characteristics is simple and practical, leading to ECCO’s recommendation of using this score for routine clinical practice [38]. 

Histologic remission is defined as NI = 0, while NI ≤ 1 defines histologic response. [37]. 

## 4. Prognostic Values for Clinical Relapse of Histological Scores

### 4.1. Geboes Score

One of the first studies to prospectively evaluate the association between Geboes grades and long-term outcomes in patients with UC was published in 2016 [39]. In this study, 179 patients with UC in clinical remission were enrolled; the baseline histologic grade was the only independent factor associated with clinical relapse over 12 months. A Geboes score > 3.1 had a sensitivity and a positive predictive value of 74% and 40%, respectively, for clinical relapse. In a Geboes score of 0 or 1, the relative risk of clinical relapse was only 0.22 [40]. 

Another study included 75 UC patients with normal endoscopy despite having basal plasmacytosis and histological inflammatory activity with a Geboes score > 3.1 (neutrophils in epithelium with >5% crypts involved) [28]. During a follow-up period of 12 months, the presence of basal plasmacytosis and a Geboes score > 3.1 was predictive of disease relapse, which was significantly higher in the group of patients with these features (37% vs. 9%). In addition, basal plasmacytosis was identified in almost 50% of the relapsers’ group. On the other side, a baseline CRP < 5 mg/L and the use of biological agents were associated with the maintenance of clinical remission. 

Histologic assessment with a Geboes score also showed excellent correlation with confocal laser endomicroscopy (CLE) when predicting relapse in patients in UC. In one study, 22 out of 23 patients with active inflammation defined as GS > 3 were also settled in the same histologic group by the evaluation of CLE [40]. A percentage of 64% of patients were classified as having active inflammation (relapsed), while only 11% in the non-active inflammation group relapsed during a follow-up of 12 months. 

### 4.2. Robarts Histopathological Index 

There is a paucity of studies reporting the sensitivity of RHI in predicting clinical relapse when compared with GS. 

This gap in the literature was addressed by one prospective study published in 2022 [41], with 187 UC patients in clinical and endoscopic remission in whom rectal biopsies were performed and histologic remission was defined as RHI < 3. Only 43% of patients with both clinical and endoscopic remission also associated with histologic remission. The risk of relapse was lower in patients with histologic remission than in those with histologic activity. The cumulative risk of relapse in patients with histologic remission at 1 and 3 years was 20.7 (vs. 45% in patients with histologic activity) and 56.3%, respectively (vs. 67.8%) [41]. Surprisingly, in a post hoc analysis [41], there was no significant difference in the risk of relapse between patients who achieved histologic remission and those with histologic activity confined only to the lamina propria [neutrophil infiltrates in the lamina propria, but not in the epithelium]. 

### 4.3. Nancy Index

There is no prospective study addressing the Nancy index (NI) as a predictor for future clinical relapse, at least to our knowledge. Several retrospective studies were identified and screened for the analysis. 

In a study published by D’Amico et al. [42], 156 UC patients were included, and approximately a quarter were considered to be in histologic remission (Nancy index 0) at baseline. Patients in histologic remission compared to those with histologic activity (defined as Nancy index > 1) underwent no surgery during follow-up (0% vs. 14%) and had a lower rate of hospitalization (7.1% vs. 36%) [42]. 

Similar results were reported in a recently published study [43] of 184 UC patients with a median follow-up of 42 months. Patients with MES 0 and histologic remission (NHI 0) had significantly higher relapse-free rates of survival. Nonetheless, after multivariate analysis, the only independent risk factor of future clinical relapse remained the histologic activity [43]. 

Due to higher clinical applicability, the Nancy index was compared to the Geboes score to prove validity in a large cohort of more than 400 UC patients [44]. NI was demonstrated to be strongly correlated with GS, with 92% of patients considered to be in histologic remission and 99% of patients considered to have histologic activity by both scores [44]. 

### 4.4. Individual Markers of Histologic Activity

In contrast to the scarcity of data regarding histologic scores as predictors of clinical relapse, there are more prospective analyses of histologic inflammatory subcomponent assessment and their correlations with the risk of relapse or exacerbation. 

Basal plasmacytosis was found to be the single histologic feature predictive of disease relapse in a prospective study of 74 patients with quiescent disease at baseline [45]. For many decades, it has been known that basal plasmacytosis can be the earliest histologic feature that advocates for the diagnosis of IBD over acute infectious colitis; in addition, beyond the scope of the study that aimed to find markers of differentiations between IBD and infectious colitis, it was also found that IBD patients who had a shorter relapse-free disease had more common basal plasmacytosis [46]. Controversially, in a more recent study, basal plasmacytosis did not appear to be a predictor of relapse [47]. 

The well-known study of Riley et al. [3] did not assess basal plasmacytosis but reported that patients with an acute inflammatory cell infiltrate (crypt abscesses and mucin depletion) and not chronic infiltrate had a higher frequency of relapse during follow-up [3]. Similar results were found in another prospective study that evaluated the role of fecal calprotectin (FC) as a predictor of relapse and histologic mucosal healing [48]; FC was predictive of disease relapse at the 6-month follow-up and histologic inflammatory activity (based on the presence of neutrophilic inflammation—cryptitis and crypt abscesses) at the 12-month follow-up [48]. Similar data were found in 113 patients with left-sided or extensive UC in clinical and endoscopic remission [15], where basal plasmacytosis acute and chronic histologic inflammatory activity were evaluated. Acute inflammatory activity (defined as the presence of intraepithelial neutrophils) remained the only risk factor for relapse within the first year of follow-up, with a risk ratio of 7.5 [15]. 

On the contrary, chronic inflammatory cell infiltrate, crypt abscesses, and basal plasmacytosis showed no statistical significance for clinical relapse in other prospective evaluations of 67 UC patients [29]; in this study, crypt architectural irregularities and mucin depletion were the only histologic abnormalities associated with relapse [29]. 

Further evidence supporting the implementation of histologic remission as a distinct target from endoscopic healing is revealed by an investigation where 91 UC patients were followed for 6 years. Histologic and not endoscopic remission predicted both corticosteroid use and acute severe colitis episodes requiring hospitalization [49]. 

The amplitude of the effect of different histologic markers on clinical outcomes has been the subject of a systematic review [50]. The absence of neutrophils in the lamina propria or epithelium predicted a lower risk of relapse or exacerbation, with an RR of 0.32 [50]. This is in contrast to the recently published post-hoc analysis of the review mentioned above, where neutrophils in the lamina propria did not influence the outcome [41]. 

Other features that predicted relapse/exacerbation were crypt abscesses, eosinophils in lamina propria, and chronic inflammatory cell infiltration. Conversely, the absence of basal plasmacytosis, basal lymphoid aggregates, and architectural crypt distortion were not associated with decreased relapse/exacerbation. Overall, histologic remission predicted a significant reduction of clinical relapse when compared to histologic activity (52% RR reduction) and a further 20% risk reduction when compared to the presence of endoscopic remission (also labeled as “macroscopic mucosal healing”) [50]. 

### 4.5. Summary of a Practical Approach

Taking into consideration the evidence presented above, we believe that a more practical approach would be beneficial from multiple perspectives. First, the homogeneity in histopathology reports that the implementation of activity scores consequently determines a better adherence in clinical practice and would support the use of histologic remission as a formal target. Secondly, the Nancy score seems to be the most simplistic approach, yet it has proven validity when compared to the more complex Geboes score. Though these scores perform well in defining histologic activity, only looking into the status of intraepithelial neutrophils also proved accurate in terms of long-term prognosis, and it represents the most accurate individual histologic marker for remission. 

## 5. Conclusions

Gastroenterologists currently focus on mucosal rather than histologic healing as a therapeutic target in ulcerative colitis. Mucosal healing is generally associated with a good prognosis, but it is not an accurate illustration of what lies underneath or within the mucosa since around one-third of patients still present inflammatory markers that predispose patients to high relapse rates. 

We admit that histologic healing is an ambitious target that requires invasive procedures, repeated biopsies and histopathologic diagnosis, parameters that secondarily have an impact in terms of financial aspects and patients’ compliance. When taking into account the proven benefits of histologic remission regarding a lower rate of relapse, hospitalizations, corticosteroid use, and also surgical risk with less need for emergency and elective colectomies, we believe that histologic healing must be considered a formal target in ulcerative colitis, at least for a particular subset of patients with unfavorable prognostic factors (i.e., younger age at diagnosis, high systemic inflammatory burden, and extensive colitis). 

Future prospective studies are needed to validate and implement the histologic scores that will hopefully settle the status of histologic remission in the next guidelines. 

## Figures and Tables

**Table 1:** The main microscopic features of inflammatory bowel disease.

Features Of Histopathological Activity	Features Of Histopathological Chronicity
Neutrophilic (or eosinophilic) cryptitis; Crypt abscesses, necrosis; Regenerative and degenerative epithelial changes; Erosions and ulcers.	Distorted crypt architecture (atrophy, foreshortening, irregular spacing and/or size of crypts); Basal plasmacytosis; Basal lymphoid aggregates; Lymphoplasmacytic infiltrate within the lamina propria; Pyloric gland metaplasia.
